# Is in vitro fertilization associated with preeclampsia? A propensity score matched study

**DOI:** 10.1186/1471-2393-14-69

**Published:** 2014-02-13

**Authors:** Noriyoshi Watanabe, Takeo Fujiwara, Tomo Suzuki, Seung Chik Jwa, Kosuke Taniguchi, Yuji Yamanobe, Kazuto Kozuka, Haruhiko Sago

**Affiliations:** 1 Department of Maternal-Fetal Neonatal Medicine, National Center for Child Health and Development, 2-10-1 Okura, Setagaya, Tokyo 157-8535, Japan; 2 Department of Social Medicine, National Research Institute for Child Health and Development, 2-10-1 Okura, Setagaya, Tokyo 157-8535, Japan; 3 Department of Information Technology and Management, National Center for Child Health and Development, 2-10-1 Okura, Setagaya, Tokyo 157-8535, Japan

**Keywords:** Blood pressure, In vitro fertilization, Preeclampsia, Propensity score, Assisted reproductive technology

## Abstract

**Background:**

Although an increased risk of preeclampsia in pregnancies conceived by in vitro fertilization (IVF) has been reported, it remains unknown whether IVF is associated with preeclampsia. In the present study, we sought to investigate whether IVF is associated with preeclampsia in pregnant women using propensity score matching analysis.

**Methods:**

This study included 3,084 pregnant women who visited the National Center for Child Health and Development before 20 weeks of gestation without hypertension or renal disease and delivered a singleton after 22 weeks of gestation between 2009 and 2011. Of the 3084 patients, 474 (15.4%) conceived by IVF (IVF group) and 2,610 (84.6%) conceived without IVF (non-IVF group). The propensity score for receiving IVF was estimated using multiple logistic regression with 27 maternal and paternal variables. This model yielded a c-statistic of 0.852, indicating a strong ability to differentiate between those conceiving with and without IVF. The association between IVF and onset of preeclampsia was assessed by the propensity matched sample (pair of N = 474).

**Results:**

There were 46 preeclampsia cases (1.5%) in the total study population, with a higher proportion of cases in the IVF group (15 cases, 3.2%) than the non-IVF group (31 cases, 1.2%). Before propensity score matching, the IVF group was 2.72 (95% confidence intervals [CI]: 1.46-5.08) times more likely to have preeclampsia when unadjusted, and 2.32 (95% CI: 1.08-4.99) times more likely to have preeclampsia when adjusted for maternal and paternal variables by logistic regression. After propensity score matching, the IVF group did not show a significantly greater association with preeclampsia compared to the non-IVF group (odds ratio: 2.50, 95% CI: 0.49-12.89), although point estimates showed a positive direction.

**Conclusions:**

Propensity score matching analysis revealed that the association between IVF and preeclampsia became weaker than when conventional adjustments are made in multivariate logistic regression analysis, suggesting that the association between IVF and preeclampsia might be confounded by residual unmeasured factors.

## Background

Preeclampsia is a major obstetric problem worldwide that causes significant maternal and perinatal morbidity and mortality [1]. Several risk factors have been identified, including advanced maternal age, primiparity, obesity, chronic hypertension, renal disease, pre-gestational diabetes mellitus, and autoimmune disease [2,3]. In addition, several studies, including a systematic review and a meta-analysis, have reported an increased risk of preeclampsia in pregnancies conceived by in vitro fertilization (IVF) [4-7]. However, these studies used limited adjustment for confounders, such as age or parity. Thus, it remains unknown whether IVF directly induces preeclampsia. A randomized control trial is the ideal study design for investigating the impact of IVF on preeclampsia, as it allows for adjusting for known and unknown confounders. However, randomized assignment of IVF treatment is impossible in studies of pregnant women.

Propensity score matching analysis is a statistical tool which can minimize selection bias and confounding factors in observational studies [8]. Estimating a propensity score for IVF treatment and matching based on the score may help determine whether IVF has a causal effect on preeclampsia. To the best of our knowledge, no study has examined the association between IVF and the incidence of preeclampsia using this statistical tool. Thus, we aimed to investigate the association between IVF and the incidence of preeclampsia by using a propensity score matching model.

## Methods

### Participants

We collected data of women who delivered between June 2009 and June 2011 at the National Center for Child Health and Development (NCCHD) in Tokyo, Japan. This study was approved by the NCCHD ethics committee. All pregnant women were Japanese. Women who delivered singleton neonates at ≥22 weeks of gestation without hypertension or renal disease were enrolled in the study. In total, we identified 3,084 women who met our study criteria (eligible study sample), including 474 women who received IVF (IVF group) and 2,610 women who did not (non-IVF group). All 3,084 study participants had only one pregnancy during this study period. Written informed consent was not obtained from participants because the study involved a secondary data analysis and such studies do not require consent according to the NCCHD ethics committee.

### Assessment of preeclampsia

Preeclampsia was diagnosed after 20 weeks of gestation if the pregnant woman had a blood pressure (BP) >140/90 mmHg with proteinuria (>300 mg/24 hours). Early-onset preeclampsia was defined as preeclampsia diagnosed at <32 weeks of gestation, and late-onset preeclampsia was defined as preeclampsia diagnosed at 32+ weeks of gestation. Severe preeclampsia was diagnosed as BP >160/110 mmHg with proteinuria (>2 g/24 hours). Women received routine prenatal checkups every four weeks up to the 28^th^ week of gestation, then every two weeks up to the 36^th^ week of gestation, and then weekly up to delivery. At each routine prenatal checkup, BP was measured in the sitting position with the right arm held at heart level after a five-minute rest period using an automated sphygmomanometer (Omron BP203RVIII oscillometer, Nippon Colin, Tokyo, Japan). Urine protein levels were assessed at each prenatal checkup by a dipstick test of a single voided urine sample. When the pregnant woman had an abnormal BP (>140/90 mmHg) and urine test (1+ proteinuria on dipstick) at a routine prenatal checkup, she was hospitalized for further evaluation of BP and urine protein and for bed rest.

### Baseline characteristics and pregnancy outcomes

Maternal characteristics, including age, age at marriage, parity, number of abortions, working status (full-time, part-time, or housewife), height, weight before pregnancy, smoking and drinking during pregnancy, family history of hypertension, mean systolic and diastolic BP before 20 weeks of gestation, and underlying diseases (central nervous system disease, cerebrovascular disease, asthma, respiratory disease other than asthma, gastrointestinal disease, liver disease, hematologic disease, cardiovascular disease, hyperthyroidism, hypothyroidism, psychiatric disease, autoimmune disease, and preexisting diabetes mellitus), were collected from the perinatal database and medical charts. Paternal factors, including age, age at marriage, height, weight, and smoking status, were also collected. Missing data for continuous variables (maternal age at marriage, 28 cases [0.9%]; maternal height, 25 cases [0.8%]; maternal weight, 26 cases [0.8%]; paternal age at delivery, 219 cases [7.1%]; paternal age at marriage, 239 cases [7.7%]; paternal height, 35 [1.1%]; and paternal weight, 35 [1.1%]) were substituted with the average value of the variable. Missing data for categorical variables were treated as dummy variables (working status, 21 cases [0.7%]; family history of hypertension, 235 cases [7.6%]; paternal smoking, 65 cases [2.1%]).

### Statistical analysis

Maternal and paternal characteristics were compared between the IVF and non-IVF groups using chi-squared or Fisher’s exact tests for categorical variables and t-tests for continuous variables.

Because IVF procedures were not randomly assigned in this population, potential confounding and selection biases were accounted for by developing a propensity score for the IVF procedure. This propensity score was determined using a multivariate logistic regression model adjusting for maternal and paternal variables. These variables included those listed above as baseline characteristics, except for drinking during pregnancy and two types of underlying diseases (central nervous system disease and cerebrovascular disease), due to their low frequency (<0.9%). Twenty-seven independent variables were thus used to determine the propensity score. This model yielded a c-statistic of 0.852, indicating a strong ability to differentiate between those conceiving with and without IVF. The 474 IVF cases were then matched one-to-n with 474 non-IVF cases with the closest propensity score (nearest neighbor matching) [9,10]. The maximum difference in propensity score between each matched pair was 0.057. Baseline characteristics and pregnancy outcomes in the eligible study sample (n = 3084) and propensity-matched study sample (n = 948) are shown in Table 1. Further, characteristics of preeclampsia (prevalence, timing of onset, and severity), BP during mid- (28-32 weeks of gestation) and late- (34-38 weeks of gestation) term, and birth outcomes in the total eligible study sample (n = 3084) and the propensity score matched sample (n = 948) were compared. For comparison, chi-squared or Fisher’s exact tests for categorical variables and t-tests for continuous variables were used for the whole study population, and conditional logistic regression for categorical variables and fixed effect regression for continuous variables were used in the propensity score matched sample.

**Table 1:** Baseline characteristics of the eligible study sample and propensity score matched sample

		**Eligible study sample (n = 3084)**			**Propensity score matched sample (n = 948)**		
		**IVF group (n = 474)**	**Non-IVF group (n = 2610)**	**P value**	**IVF group (n = 474)**	**Non-IVF group (n = 474)**	**P value**
**Maternal demographics**							
Age	Year, mean (SD)	38.3 (3.3)	34.8 (4.2)	**<0.001**	38.3 (3.3)	38.4 (3.3)	0.97
Age at marriage	Year, mean (SD)	30.8 (4.1)	29.3 (3.8)	**<0.001**	30.8 (4.1)	31.0 (4.1)	0.91
Parity	0, n (%)	370 (78.1)	1319 (50.5)	**<0.001**	370 (78.1)	378 (79.8)	0.74
	≥1, n (%)	104 (21.9)	1291 (49.5)		104 (21.9)	96 (20.3)	
Abortion	0, n (%)	291 (61.4)	1767 (67.7)	**0.009**	291 (61.4)	304 (64.1)	0.39
	1-2, n (%)	152 (32.1)	734 (28.1)		152 (32.1)	136 (28.7)	
	3≤, n (%)	31 (6.5)	109 (4.2)		31 (6.5)	34 (7.2)	
Work	Homemaker, n (%)	189 (39.9)	1122 (43.0)	0.31	189 (39.9)	190 (40.1)	0.93
	Full time, n (%)	248 (52.3)	1268 (48.6)		248 (52.3)	238 (50.2)	
	Part time, n (%)	36 (7.6)	200 (7.7)		36 (7.6)	45 (9.5)	
	Missing, n (%)	1 (0.2)	20 (0.8)		1 (0.2)	1 (0.2)	
**Maternal health status**							
Height	cm, mean (SD)	160.0 (5.4)	159.4 (5.2)	**0.015**	160.0 (5.4)	159.8 (5.1)	0.75
Weight before pregnancy	kg, mean (SD)	52.4 (6.9)	51.5 (7.8)	**0.011**	52.4 (6.9)	52.0 (7.2)	0.74
Smoking during pregnancy	Yes, n (%)	6 (1.3)	92 (3.5)	**0.01**	6 (1.3)	6 (1.3)	0.27
Drinking during pregnancy	Yes, n (%)	0 (0)	11 (0.4)	0.39	0 (0)	0 (0)	NA
Family history of hypertension	Yes, n (%)	110 (23.2)	414 (15.9)	**<0.001**	110 (23.2)	104 (21.9)	0.82
Systolic blood pressure <20 weeks of gestation	mmHg, mean (SD)	110.4 (10.4)	108.4 (10.2)	**<0.001**	110.4 (10.4)	110.9 (9.9)	0.75
Diastolic blood pressure <20 weeks of gestation	mmHg, mean (SD)	64.8 (7.3)	62.8 (7.1)	**<0.001**	64.8 (7.3)	65.0 (7.0)	0.86
**Maternal medical complication**							
Central nervous system disease	Yes, n (%)	0 (0)	27 (1.0)	**0.016**	0 (0)	1 (0.2)	>0.99
Cerebrovascular disease	Yes, n (%)	0 (0)	4 (0.2)	>0.99	0 (0)	1 (0.2)	>0.99
Asthma	Yes, n (%)	13 (2.7)	92 (3.5)	0.49	13 (2.7)	9 (1.9)	0.29
Respiratory disease (except asthma)	Yes, n (%)	2 (0.4)	3 (0.1)	0.17	2 (0.4)	4 (0.8)	>0.99
Gastrointestinal disease	Yes, n (%)	2 (0.4)	33 (1.3)	0.15	2 (0.4)	1 (0.2)	0.57
Liver disease	Yes, n (%)	3 (0.6)	16 (0.6)	>0.99	3 (0.6)	2 (0.4)	0.57
Hematologic disease	Yes, n (%)	2 (0.4)	16 (0.6)	>0.99	2 (0.4)	2 (0.4)	>0.99
Cardiovascular disease	Yes, n (%)	5 (1.1)	51 (2.0)	0.26	5 (1.1)	4 (0.8)	0.42
Hyperthyroidism	Yes, n (%)	7 (1.5)	47 (1.8)	0.85	7 (1.5)	4 (0.8)	>0.99
Hypothyroidism	Yes, n (%)	13 (2.7)	70 (2.7)	0.88	13 (2.7)	14 (3.0)	0.29
Psychiatric disease	Yes, n (%)	10 (2.1)	64 (2.5)	0.75	10 (2.1)	9 (1.9)	>0.99
Autoimmune disease	Yes, n (%)	9 (1.9)	55 (2.1)	0.86	9 (1.9)	6 (1.3)	>0.99
Pre-existing diabetes mellitus	Yes, n (%)	1 (0.2)	15 (0.6)	0.49	1 (0.2)	2 (0.4)	0.57
**Paternal demographics**							
Age	Year, mean (SD)	40.8 (4.8)	37.3 (4.9)	**<0.001**	40.8 (4.8)	40.6 (4.8)	0.25
Age at marriage	Year, mean (SD)	33.3 (5.3)	31.7 (4.8)	**<0.001**	33.3 (5.3)	33.2 (5.6)	0.37
**Paternal health status**							
Partner's smoking status	Yes, n (%)	136 (28.7)	705 (27.0)	0.31	136 (28.7)	140 (29.5)	0.84
	Missing, n (%)	6 (1.3)	59 (2.3)		6 (1.3)	6 (1.3)	
Partner's height	cm, mean (SD)	172.7 (5.8)	173.1 (5.7)	0.16	172.7 (5.8)	173.4 (5.6)	0.10
Partner's weight	kg, mean (SD)	69.6 (9.2)	69.8 (9.6)	0.70	69.6 (9.2)	70.1 (9.0)	0.64

IVF, in vitro fertilization; SD, standard deviation.

Chi-squared or Fisher’s exact tests for categorical variables and t-tests for continuous variables were used for the whole study population. Conditional logistic regression for categorical variables and regression analysis using the fixed effect model for continuous variables were used in the propensity score matched sample.

Values in bold are significant at the p = 0.05 level.

To assess the association between IVF and preeclampsia, we performed a logistic regression analysis in the initial eligible study sample (n = 3084), unadjusted and adjusted for baseline characteristics, and performed a conditional logistic regression analysis for the propensity score matched sample (n = 948). All analyses were conducted using STATA MP software (version 12.0; Stata Corporation, College Station, TX).

## Results

A comparison of baseline characteristics and pregnancy outcomes between the IVF (n = 474) and non-IVF (n = 2,610) groups is shown in Table 1. Women in the IVF group were older than those in the non-IVF group at the time of first visit (38.3 vs. 34.8 years, p < 0.001) and at marriage (30.8 vs. 29.3 years, p < 0.001). Women in the IVF group were more likely to be nulliparous (78.1 vs. 50.5%, p < 0.001) and to have had abortions than those in the non-IVF group (38.6 vs. 32.3%, p = 0.009). Women in the IVF group were slightly taller and weighed more than those in the non-IVF group (height, 160.0 vs. 159.4 cm, p = 0.015; weight, 52.4 vs. 51.5 kg, p = 0.011). Furthermore, women in the IVF group were less likely to smoke (1.3 vs. 3.5%, p = 0.01) but more likely to have a family history of hypertension (23.2 vs. 15.9%, p < 0.001). Mean BP before 20 weeks of gestation, which is considered a proxy of baseline BP, was higher in the IVF group than in the non-IVF group for both systolic (110.4 vs. 108.4 mmHg, p < 0.001) and diastolic (64.8 vs. 62.8 mmHg, p < 0.001) BP. The prevalence of underlying disease was not significantly different between the IVF and non-IVF groups, except for central nervous system disease. Partners of women in the IVF group were older than those of women in the non-IVF group at the time of first visit (40.8 vs. 37.3 years, p < 0.001) and at marriage (33.5 vs. 31.7 years, p < 0.001).

Baseline characteristics of the IVF and non-IVF groups after propensity score matching (each n = 474) were not significantly different. For example, maternal age for the IVF and non-IVF groups was 38.3 and 38.4 years (p = 0.97), respectively, and mean diastolic BP was 64.8 and 65.0 mmHg (p = 0.86) for the IVF and non-IVF groups, respectively.

Table 2 shows the characteristics of preeclampsia and BP during mid- and late-pregnancy in the IVF (n = 474) and non-IVF (n = 2,610) groups. Preeclampsia was found in 15 (3.2%) cases in the IVF group and 31 (1.2%) cases in the non-IVF group. Among those who had preeclampsia, the number of early onset (<32 weeks of gestation) cases was two (13.3%) for the IVF group and three (9.7%) for the non-IVF group, which was not significantly different (p = 0.71). Further, the severity of preeclampsia was not different between the IVF and non-IVF groups: severe preeclampsia was found in eight (53.3%) cases in the IVF group and 17 (54.8%) cases in the non-IVF group (p = 0.92). Mean diastolic BP during mid- and late-pregnancy was significantly higher in the IVF group than in the non-IVF group (mid-pregnancy, 64.5 vs. 62.9 mmHg, p < 0.001; late-pregnancy, 67.4 vs. 65.9 mmHg, p < 0.001), while systolic BP during mid- and late-pregnancy were not significantly different. Birth weight was higher in the IVF group than in the non-IVF group (3042.7 vs. 2988.1 g, p = 0.008), while gestational age was not significantly different.

**Table 2:** Characteristics of preeclampsia and blood pressure during mid- and late-pregnancy

		**Eligible study sample (n = 3084)**			**Propensity score matched sample (n = 948)**		
		**IVF group (n = 474)**	**Non-IVF group (n = 2610)**	**P value**	**IVF group (n = 474)**	**Non-IVF group (n = 474)**	**P value**
**Preeclampsia**^a^							
Prevalence	n (%)	15 (3.2)	31 (1.2)	**0.003**	15 (3.2)	7 (1.5)	0.27
Timing (among those with preeclampsia)							
Early onset (<32 weeks of gestation)	n (%)	2 (13.3)	3 (9.7)	0.71	2 (13.3)	0 (0)	0.27
Late onset (≥32 weeks of gestation)	n (%)	13 (86.7)	28 (90.3)		13 (86.7)	7 (100)	
Severity (among those with preeclampsia)							
Mild	n (%)	7 (46.7)	14 (45.2)	0.92	7 (46.7)	3 (42.9)	0.27
Severe	n (%)	8 (53.3)	17 (54.8)		8 (53.3)	4 (57.1)	
**Blood pressure**							
SBP during mid-pregnancy^b^	mmHg, mean (SD)	109.0 (9.6)	108.2 (9.7)	0.10	109.0 (9.6)	109.3 (10.1)	0.42
DBP during mid-pregnancy^b^	mmHg, mean (SD)	64.5 (6.6)	62.9 (6.9)	**<0.001**	64.5 (6.6)	64.3 (7.3)	0.99
SBP during late-pregnancy^c^	mmHg, mean (SD)	112.1 (9.6)	111.2 (9.7)	0.068	112.1 (9.6)	112.1 (9.0)	0.35
DBP during late-pregnancy^c^	mmHg, mean (SD)	67.4 (6.7)	65.9 (7.0)	**<0.001**	67.4 (6.7)	67.1 (6.8)	0.69
**Birth outcomes**							
Birth weight	g, mean (SD)	3042.7 (425.7)	2988.1 (408.0)	**0.008**	3042.7 (425.7)	3007.0 (414.0)	0.21
Gestational age	weeks, mean (SD)	39.2 (1.6)	39.1 (1.6)	0.15	39.2 (1.6)	39.2 (1.7)	0.77

^a^Preeclampsia is considered severe if systolic blood pressure (BP) is ≥160 mmHg or diastolic BP is ≥110 mmHg on two occasions at least six hours apart while the patient is on bed rest, or if proteinuria is ≥2 g/24 hours or ≥3 on dipstick in two random urine samples collected at least four hours apart.

^b^mid-pregnancy: 28-32 weeks of gestation.

^c^late-pregnancy: 34-38 weeks of gestation.

IVF, in vitro fertilization; SD, standard deviation; SBP, systolic blood pressure; DBP, diastolic blood pressure.

Chi-squared or Fisher’s exact tests for categorical variables and t-tests for continuous variables were used for the whole study population. Conditional logistic regression for categorical variables and regression analysis using the fixed effect model for continuous variables were used in the propensity score matched sample.

Values in bold are significant at the p = 0.05 level.

Table 2 also shows the characteristics of preeclampsia and BP during mid- and late-pregnancy in the propensity score matched sample (each n = 474). Although the prevalence of preeclampsia in the IVF group was higher than in the non-IVF group (3.2% vs. 1.5%), the difference was not significant after propensity matching (p = 0.27). Further, mean diastolic BP during mid- and late-pregnancy was not significantly different (mid-pregnancy, 64.5 vs. 64.3 mmHg, p = 0.99; late-pregnancy, 67.4 vs. 67.1 mmHg, p = 0.69). Birth weight also did not significantly differ.

Table 3 shows the odds ratios (ORs) of IVF for the risk of preeclampsia in the total eligible study sample (n = 3,084), unadjusted and adjusted for baseline characteristics, and propensity score matched sample (each n = 474). In the unadjusted model using the eligible study sample, IVF was a significant risk factor for preeclampsia (OR: 2.72, 95% confidence interval [CI]: 1.46–5.08, p = 0.002). After adjusting for baseline maternal and paternal characteristics, IVF was still significantly associated with preeclampsia (OR: 2.32; 95% CI: 1.08–4.99, p = 0.031). However, in the propensity score matched sample, IVF was not significantly associated with preeclampsia, although the point estimation of the risk of IVF for preeclampsia was in the same positive direction as with the previous models (OR: 2.50, 95% CI: 0.49-12.89, p = 0.273).

**Table 3:** Odds ratio of developing preeclampsia among patients who had IVF

**Model**	**Odds ratio (95% CI)**	**P value**
Eligible study sample, unadjusted (n = 3084)	**2.72 (1.46-5.08)**	**0.002**
Eligible study sample, adjusted for baseline characteristics^a^ (n = 3084)	**2.32 (1.08-4.99)**	**0.031**
Propensity score matched sample (pair of n = 474)	2.50 (0.49-12.89)	0.273

^a^adjusted confounders were maternal characteristics (age, age at marriage, parity, number of abortions, working status, height, weight before pregnancy, smoking and drinking during pregnancy, family history of hypertension, mean systolic and diastolic blood pressure before 20-weeks gestational age, and underlying diseases such as central nervous system disease, cerebrovascular disease, asthma, hyperthyroidism, hypothyroidism, psychiatric disease, and autoimmune disease) and paternal characteristics (age, age at marriage, height, weight, and smoking status).

Multiple logistic regression was used for analysis.

Values in bold are significant at the p = 0.05 level.

## Discussion

In the present study, we used propensity score matching analysis and adjusted for 27 known variables to simulate random assignment of the IVF procedure. Using this analysis, we found that women who conceived by IVF were not at a significantly higher risk of preeclampsia than women who conceived without IVF, although the point estimation of the risk of IVF for preeclampsia was positive (i.e. 2.50), suggesting that IVF may be weakly associated with preeclampsia.

To the best of our knowledge, this is the first study to investigate the impact of IVF on preeclampsia using propensity score matching analysis. In a 2004 meta-analysis of eight studies, Jackson et al. concluded that IVF was associated with an increased risk of preeclampsia (OR: 1.55; 95% CI: 1.23–1.95) [4], although the point estimate of OR was lower than that of our study, which might be due to ethnic differences of the sample, or by chance. Moreover, the studies used in the meta-analysis adjusted for only a limited number of confounders, such as age and parity, thereby limiting the ability to adjust for assignment bias in the estimated effect. After this meta-analysis, several investigators demonstrated associations between IVF and preeclampsia [5,6,11]. Shevell et al. adjusted for more confounders (age, race, marital status, years of education, prior preterm delivery with anomaly, body mass index [BMI], smoking history, and bleeding during the current pregnancy), and reported a significant association between IVF and preeclampsia (OR: 2.7, 95% CI: 1.7–4.4) [5]. Our study, which used propensity score matching with 27 variables, also showed a marginal impact of IVF on preeclampsia, which is an important contribution to the literature.

Specifically, we demonstrated that IVF was not significantly associated with preeclampsia, although the point estimate of OR was 2.50, which implies that IVF is a risk factor for preeclampsia. The lack of a significant association might be due to the small sample size (n = 948); however, attenuation of the point estimate of OR from the unadjusted model (i.e., 2.72) to the propensity score analysis (2.50) was substantial, suggesting that the association observed between IVF and preeclampsia in previous studies might have been due to confounders.

Further, the timing of onset of preeclampsia, severity of preeclampsia, and BP during mid- and late-pregnancy did not differ between the IVF and non-IVF groups. These results suggest that IVF may not elevate BP or induce preeclampsia because women with preeclampsia have higher BP levels throughout pregnancy [12]. Macdonald-Wallis et al. reported that age, BMI, and parity affected BP throughout pregnancy [13], but no single risk factor elevated BP during only early pregnancy.

A plausible biological mechanism to explain how there could be a causal relationship between IVF and preeclampsia is unclear. Abnormal placentation is considered the primary stage of pathogenesis in preeclampsia [14]. In a recent systematic review, several biological mechanisms by which IVF may be associated with preeclampsia by abnormal placentation have been proposed [7]. Transfer of the conceptus into the uterine cavity and the effect of an altered hormonal environment in the uterine myometrium during the IVF procedure may interfere with the development of the maternal-fetal interface. Additionally, as the formation of the chorion is initiated in vitro in IVF pregnancies, the inherent difference in the nature of the placenta may lead to abnormal placentation and diseased placental vessels. Furthermore, inadequate utero-placental circulation may contribute to the association between IVF and preeclampsia.

There are several limitations to our study. First, given our observational study design, we could not completely avoid selection biases and unknown confounding factors (e.g., personality and genetic traits) that existed before IVF treatment. These factors may have affected pregnancy outcomes, including the incidence of preeclampsia, despite adjusting for 27 variables using a propensity score. Moreover, as we used secondary data, assessment of subfertility was not possible, although it was reported to be associated with an increased risk of preeclampsia [15,16]. To overcome this limitation, a randomized controlled trial would be ideal, but not feasible. Future studies will need to investigate additional variables in order to better estimate the propensity score for undergoing IVF. Second, our study was performed at a single tertiary perinatal center, although the IVF patients were referred to the center from other fertility clinics. Finally, our study only included pregnant Japanese women. As such, we could not assess the effect of race on the association between IVF and preeclampsia.

In conclusion, we found that IVF was weakly associated with preeclampsia when using propensity score matching analysis (which provides a better estimate of causality than adjusting for confounders) than when using simple adjustments in multivariate logistic regression analysis. This suggests that the influence of the IVF procedure itself was weaker than observed in previous studies. Future studies with a larger study population that can assess more confounding variables are needed to replicate our findings.

## Conclusions

Propensity score matching analysis revealed that the association between IVF and preeclampsia became weaker than with conventional adjustments in a multivariate logistic regression, suggesting that the association between IVF and preeclampsia might be confounded by residual unmeasured factors.

## Abbreviations

IVF: In vitro fertilization; BP: Blood pressure; OR: Odds ratios; CI: Confidence interval.

## Competing interests

The authors report no conflicts of interest. The authors alone are responsible for the content and writing of the paper.

## Authors’ contributions

WN and FT conceived and designed the study. WN, ST, TK, YY, KK and SH contributed to clinical data collection. WN and FT contributed to statistical analysis and interpretation of data. WN drafted the manuscript and TF finalized it. All authors contributed to critical revision and final approval of the manuscript.

## Pre-publication history

The pre-publication history for this paper can be accessed here:

http://www.biomedcentral.com/1471-2393/14/69/prepub
